# The distribution shifts of *Pinus armandii* and its response to temperature and precipitation in China

**DOI:** 10.7717/peerj.3807

**Published:** 2017-09-15

**Authors:** Xiaofeng Zheng, Pengxiang Gao, ShuoXin Zhang

**Affiliations:** 1 College of Forestry, Northwest A&F University, Yangling, Shaanxi, China; 2 Qinling National Forest Ecosystem Research Station, Northwest A&F University, Yangling, Shaanxi, China

**Keywords:** Elevation, *Pinus armandii*, Tree distribution shift, Precipitation, Changing climate, Temperature

## Abstract

**Background:**

The changing climate, particularly in regard to temperature and precipitation, is already affecting tree species’ distributions. *Pinus armandii,* which dominates on the Yungui Plateau and in the Qinba Mountains in China, is of economic, cultural and ecological value. We wish to test the correlations between the distribution shift of *P. armandii* and changing climate, and figure out how it tracks future climate change.

**Methods:**

We sampled the surface soil at sites throughout the distribution of *P. armandii* to compare the relative abundance of pollen to the current percent cover of plant species. This was used to determine possible changes in the distribution *P. armandii*. Given the hilly terrain, elevation was considered together with temperature and precipitation as variables correlated with distribution shifts of *P. armandii*.

**Results:**

We show that *P. armandii* is undergoing change in its geographic range, including retraction, a shift to more northern areas and from the upper high part of the mountains to a lower-altitude part in hilly areas. Temperature was the strongest correlate of this distribution shift. Elevation and precipitation were also both significantly correlated with distribution change of *P. armandii*, but to a lesser degree than temperature.

**Conclusion:**

The geographic range of *P. armandii* has been gradually decreasing under the influence of climate change. This provides evidence of the effect of climate change on trees at the species level and suggests that at least some species will have a limited ability to track the changing climate.

## Introduction

Multiple lines of scientific study show that climate change strongly and rapidly affects the global ecosystem (Walther et al., 2002; Peñuelas & Filella, 2001), as well as animals and plants (Root et al., 2003). Forests in particular are an important part of the terrestrial ecosystem that are sensitive to climate change (Aber et al., 2001). Climate change will inevitably have an impact on forests, and considerable changes will occur in forests due to climate change (Parmesan, 2006). Climate change, including increasing temperature and precipitation, has also been identified as the primary regulator of plant distribution (Box, 1981) in altitudinal ranges (Parmesan, 2006; Groom, 2013).

*P. armandii* is a pine species native to China that occurs in southern Shaanxi, southern Gansu, western Sichuan, Yunnan and western Guizhou provinces. Its timber can be used for general building purposes, and the nuts and pollen powder are also of great economic value. *P. armandii* is the dominant pine tree species, and one of the most widely distributed tree species in natural forests and important forestry plantations in these areas. Obvious changes in temperature and precipitation have been observed and reported in these areas based on the climate data of the China Meteorological Administration; some researchers have cited these changes in their publications (Wang et al., 2004; Song, Yan & Ma, 2011; You, He & Duan, 2005; Ren et al., 2005). Forests are faced with the threat of deforestation (Malhi et al., 2008) and/or migration, starting with changes in the distribution of other tree species.

Climate-environment relationship models for different scenarios have been established to quantify the current influence of climate on plant distribution and to forecast their evolution (Heikkinen et al., 2006; Pearson et al., 2006) based on rigorous data and thorough validation assessments. Hutchinson (1957) developed a bioclimate envelope to define climatic components of an ecological niche that includes all the environmental variables affecting a species. The Spatial Evaluator of Climate Impacts on the Envelope of Species (SPECIES), by Pearson, Dawson & Berry (2002), employs an artificial neural network (ANN) to characterize bioclimate envelopes based on observed species distributions and five environmental inputs (derived primarily from climatic data and including a measure of the soil type). These ecosystem-climate modeling approaches require thoroughly assessed validation and strong databases, which makes it very difficult to employ the relevant models in regions with poor databases.

Surface pollen analysis essentially reflects the modern vegetation composition (Minckley & Whitlock, 2000), to some extent based on the pollen-vegetation calibration. Luo et al. (2009) presented a modern pollen–vegetation dataset based on surface soil samples in the mountainous areas of China. Their results indicated that surface pollen was correlated with the dominant vegetation community in the study regions. Understanding the relationship between modern pollen and vegetation is of great significance in detecting the impacts of climate on regional vegetation shift, including both temperature and precipitation (Zhang et al., 2010).

Hence, based on the surface pollen analysis, we were interested in the following:

1. Whether the distribution of *P. armandii* has been changing.
2. If it has been changing, what environmental variables are correlated with this change.
3. A rough forecast of future change in distribution.

Studying change in the geographic and altitudinal distribution of tree species in a mountain forest ecosystem in response to climate factors, mainly temperature and precipitation is meaningful for our understanding of both forests and the global ecosystem. We hope that our study of the distribution of *P. armandii* contributes to future studies on ecosystem structure and community succession.

## Methods

### Study area

**Figure 1:**
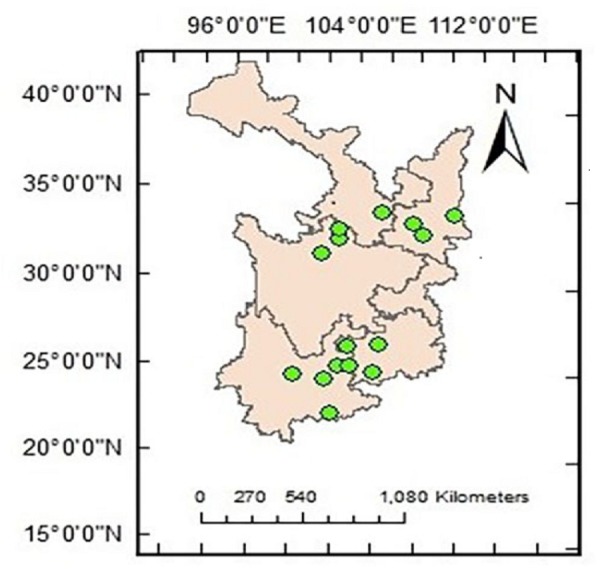
Location of the study locations.

The study area ranges from 101°33′ to 108°21′E and from 22°59′ to 34°34′N, encompassing the Yungui Plateau and the Qinba Mountains and including Shaanxi, Gansu, Sichuan, Yunnan and Guizhou provinces in the eastern part of western China. The Qinling Mountains consist of a deciduous forest region (Olson et al., 2001) across the 800-mm precipitation line of China. The common forest species are *Pinus, Quercus, Picea, Ulmus, Larix, Acer, Fraxinus, Castanopsis, Celtis, Betula, Carpinus,* and *Abies,* among others*.* The rainy climate and high altitude of the Yungui Plateau result in dense forests. Coniferous species such as *Pinus, Larix, Tsuga, Picea,* and *Abies* and evergreen families such as Magnoliaceae, Hamamelidaceae, Lauraceae, Theaceae, and Fagaceae are all widely distributed. Fifteen locations were determined to contain a natural *Pinus* distribution (Fig. 1).

### Field work

The detailed sampling features of each location are shown in Table 1. Most of the forests are in mountainous areas, so we performed distribution-centered vertical sampling. Surface pollen samplings were collected from all of the 297,20 × 20 m plots chosen by the natural distribution. The samples originated from pure and mixed forests in which *Pinus* is in or around. We chose the sampling point to be the center of the corresponding plot. All samplings occurred away from roadside, where human disturbance was negligible. The plant coverage of *Pinus* was calculated separately in each plot, using the number of *Pinus* trees divided by total arbor number, including shrubs higher than 2 m.

**Table 1:** Characteristics of each study location.

Location	Number of plot	Province	Coordinate	Coverage range (elevation)
Maiji mountain	15	Gansu	34°34′N 105°52′E	1,200 m–1,700 m
Taibai Mountain	15	Shaanxi	33°57′N 107°45′E	1,500 m–2,300 m
Huoditang	24	Shaanxi	33°18′N 108°21′E	1,500 m–2,100 m
Mount Hua	20	Shaanxi	34°25′N 109°57′E	1,200 m–1,800 m
Jiuzhaigou	15	Sichuan	32°54′N 103°46′E	2,000 m–2,200 m
Aba	15	Sichuan	32°01′N 102°34′E	2,100 m–2,300 m
Zoige	9	Sichuan	33°29′N 103°31′E	2,400 m–2,500 m
Chuxiong	24	Yunnan	25°02′N 101°33′E	1,000 m–2,800 m
Pingbian	24	Yunnan	22°59′N 103°41′E	800 m–1,400 m
Zhanyi	24	Yunnan	25°41′N 103°50′E	1,000 m–2,100 m
Yiliang	16	Yunnan	24°55′N 103°11′E	1,000 m–3,400 m
Weining	24	Guizhou	26°52′N 104°17′E	1,400 m–2,300 m
Panxian	24	Guizhou	25°43′N 104°28′E	1,000 m–2,500 m
Qianxi	24	Guizhou	27°02′N 106°01′E	1,200 m–1,600 m
Ziyun	24	Guizhou	25°26′N 105°46′E	1,000 m–1,400 m

### Laboratory and data analysis

*Pinus* has an overrepresented pollen type, which means that the percentage of pollen is larger than that of the plant cover; however, its pollen percentage is still related to plant cover (Xu et al., 2007). According to Li & Yao (1990), *Pinus* individuals should be found in plots where the percentage of *Pinus* surface pollen is higher than 30%, considering the *Pinus* pollen dispersal kernels, including wind and other population sources, which is to say: “Plant Cover = 0” + “Surface Pollen Percentage > 30%” = “a post retreat”. Comparing the surface pollen analysis with the results of the vegetation investigation could reveal the status of distribution/coverage.

Twenty grams of soil was weighed to extract pollen. The pollen analysis method followed that of Faegri, Kaland & Krzywinski (1989). The specific steps involved taking *Lycopodium* (27,637 ± 563 grains) liquid for tracers, using a 200-µm strainer to remove the larger plant fragments or other impurities, adding 10% HCl and 10% NaOH solutions and, finally, floating the pollen in a specific-gravity solution (1.9–2.2). Only the *Pinus* pollen was identified and counted using a 400×-magnification light microscope. The pollen percentage was calculated using the number of *Lycopodium* grains as a base value in this study.

Based on the collected climate data, the regular patterns of mean annual precipitation and mean annual temperature in the distribution areas of *P. armandii* in its naturally occurring distribution regions could help identify the main driver leading the change. Ordination analysis reveals the relationship of environmental factors and *P. armandii* distribution locations and can potentially be used to forecast the future distribution of *P. armandii*.

Principal component analysis was conducted to explain the contributions of each climate parameter to environmental variation among the sites. Most sampling districts were hilly terrain where the climate conditions, including temperature and precipitation, are clearly affected throughout the change in elevation (Giorgi et al., 1997). We took elevation together with temperature and precipitation as an explanatory factor parameter in this study and explored its regional influence. The average temperature and precipitation data within the study area were collected from the local weather bureau or meteorological station. Ordination analysis was conducted to characterise the relationship between the environmental parameters and the individual study areas; specifically, redundancy analysis (RDA) was conducted due to the short gradient lengths calculated by the detrended correspondence analysis (DCA). R version 3.3.3 (R Core Team, 2017) and Canoco for Windows 4.5 (Ter Braak & Smilauer, 2002) were used to perform the data analysis and to construct the figures.

## Results

### Change in the distribution of *P. armandii*

**Figure 2:**
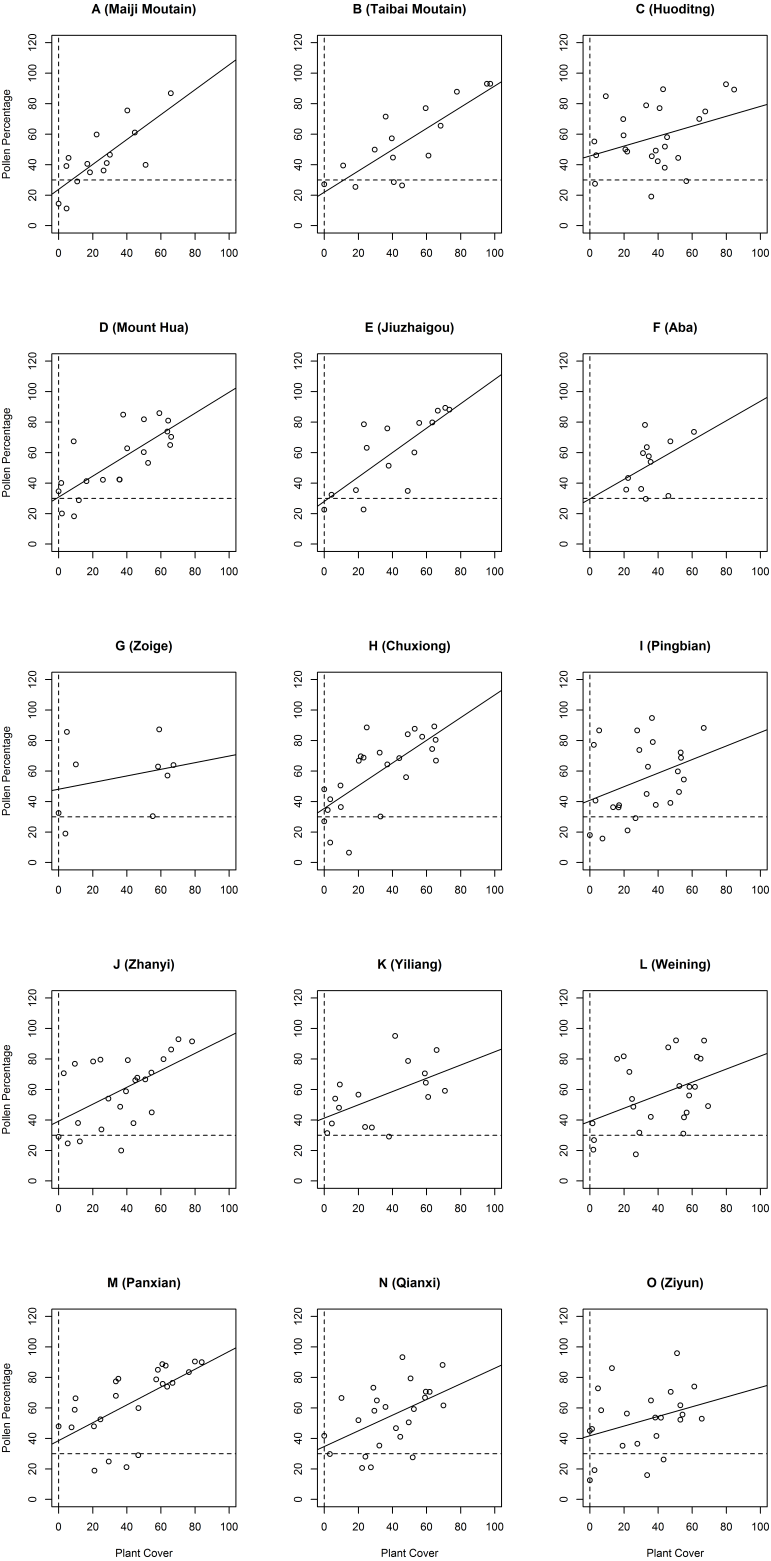
The pollen percentage and plant cover. *X* axis represents the *Pinus* tree cover; *Y* axis represents the *Pinus* pollen percentage. Sites with no *Pinus* trees found during the investigation are located on the vertical dotted line. The horizontal dotted line is the 30% *Pinus* pollen percentage line, which is the boundary line. If the no-*Pinus*-tree sites are beyond the boundary line, a decline of *Pinus* distribution can be inferred.

We investigated and organized the main types of *Pinus* forests of the Qinba Mountains and Yungui Plateau; the results are shown in Text S1. In the field investigation, we found many samples with no *Pinus* trees, despite the *Pinus* pollen percentage being higher than 30% in all the sampling districts. The relationship between *Pinus* pollen percentage and *Pinus* cover is shown in Fig. 2, categorized by regions. 11 (out of 15) intercepts of the regression lines on the *Y* axis of the *Pinus* percentage and plant cover regions are higher than 30%, except for those of Maiji Mountain, Taibai Mountain, Jiuzhaigou and Aba; these study regions had intercepts lower than 30%. Huoditang and Zoige had the largest intercepts, even higher than 40%. The results indicate that *P. armandii* cover has undergone a distributional decline or a general shift in these regions. The distribution dispersal status on Maiji Mountain and Taibai Mountain, as well as in Jiuzhaigou and Aba, were unclear.

### Environmental correlates of change in the distribution of *P. armandii*

The results of principal component analysis demonstrating the regional influence of climate factors are shown in Table 2. Mean annual temperature, elevation and mean annual precipitation all seem to impact the distribution shift *of P. armandii*. Mean annual temperature is the primary environmental factor correlated with distribution change across the study areas, followed by elevation, then mean annual precipitation. Mean annual temperature was the most important correlate of change in the distribution of *P. armandii*, with a proportion up to 67.72%, while elevation and mean annual precipitation were 19.80% and 12.48%.

**Table 2:** Principal components analysis results.

Component	Initial eigenvalues			Extraction sums of squared loadings	
	Total	% of variance	Cumulative (%)	Total	% of variance
Temperature	2.03	67.72	67.72	2.03	67.72
Elevation	0.59	19.80	87.52	0.59	19.80
Precipitation	0.37	12.48	100	0.37	12.48

### A rough forecast of change in the distribution of *P. armandii*

**Figure 3:**
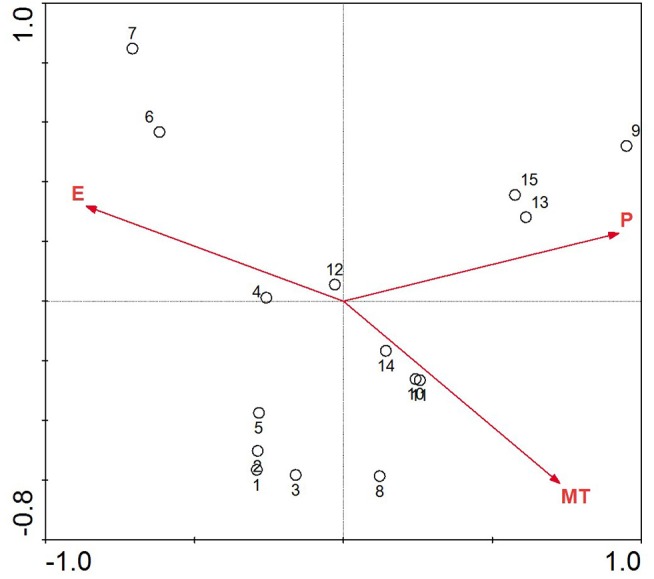
Redundancy analysis ordination biplot showing the relationship between environmental variables and location. The points with Arabic numerals represent the location, and the arrow lines with capital letters represent the environmental parameter (1, Maiji Mountain; 2, Taibai Mountain; 3, Huoditang; 4, Mount Hua; 5, Jiuzhaigou; 6, Aba; 7, Zoige; 8, Chuxiong; 9, Pingbian; 10, Zhanyi; 11, Yiliang; 12, Weining; 13, Panxian; 14, Qianxi; 15, Ziyun; P, mean annual precipitation; MT, mean annual temperature; E, elevation).

The DCA of the location and environment in Table 3 helped us choose the ordination analysis method. The first two eigenvalues of the DCA are high, implying that the first two axes represent a strong gradient, while the third and fourth axes are much weaker. All axis gradient lengths in the results from the location-environment DCA were less than 3, so RDA was chosen for the ordination. The results of the RDA of the climate parameters and the study districts are shown in Fig. 3. The mean annual precipitation and mean annual temperature increases are from left to right along the first axis of the redundancy analysis ordination diagram, while the elevation decreases concurrently.

**Table 3:** Location-environment DCA.

Axes	1	2	3	4	Total inertia
Eigenvalues	0.26	0.32	0.01	0.01	0.58
Lengths of gradient	0.69	0.69	0.62	0.54	
Location-environment correlations	0.99	0.99	0	0	
Cumulative percentage variance:					
of location data	98.50	99.10	100	100	
of location-environment relation	99.40	100	0	0	

Along the direction-of-temperature arrow, the majority locations appear, except Aba and Zoige; the narrow biotope with high elevation in these two locations makes them different (Table 1). The vertical projects of other sample locations on the temperature variable line are close and concentrated; these samples will be intensively influenced by the mean annual temperature. *P. armandii* in these study locations will be strongly affected by changing temperature, and along the temperature arrow, increasing temperature will limit the distribution in future. On the precipitation and elevation line, the vertical projects are well distributed. *P. armandii* in the Pingbian, Panxian and Ziyun will be challenged intensely as the precipitation increases, and *P. armandii* in Maiji Mountain is prone to increasing precipitation. As to elevation, Aba and Zoige are more sensitive to high elevation, while Pingbian is more sensitive to low elevation. These results can be explained by the narrow elevation coverage and relatively low elevation scope (Table 1). *P. armandii* in the rest of the study locations have different capacity to the current precipitation status and elevation range. Both elevation and mean annual precipitation have an even impact power on these sites, unlike the mean annual temperature.

## Discussion

In this study, we verified that the distribution of *Pinus armandii* will tend to shrink under the influence of climate change in the future. A study in America demonstrated the existence of an impact at the tree species level: *P. albicaulis* will be extirpated from most of its current range as temperatures rise (McLane & Aitken, 2012). Temperature is one of the most important factors controlling the change of geographical ranges of species (Ellenberg, 1988). Studies that have employed bioclimatic envelope models to estimate species distribution changes also show that temperature has determined plant species distributions (Hirota et al., 2011; Toledo, 2012). Thus, increased temperatures due to climate change are likely to have been causing tree distribution shifts to higher elevations (Tewksbury, Sheldon & Ettinger, 2011) similar to that observed in this study. Further, elevation is correlated with temperature in that lower elevation and lower latitude sites are warmer. Therefore, a much stronger impact by increasing temperature on tree distribution shift is expected. This result is in line with the main evidence of the ecological impacts of climate change. Climate change has been demonstrated to be a strong factor on tree distribution (Canham & Thomas, 2010), which means that climate change will have a profound influence on the range of expansion and contraction (Walther et al., 2002). As a part of the forest ecosystem, *P. armandii* is influenced by the climate.

When exploring the main factors affecting shifts in species distributions due to climate change, some scientific researchers have focused on the warming climate, i.e., temperature (Beckage, Osborne & Gavin, 2008), and some have considered both temperature and precipitation (Kelly & Goulden, 2008). The main causal factors may differ by ecological niche. Our results suggest that precipitation also influences the distribution shift of *Pinus,* though in a supplementary, rather than primary, way. The rare high mean annual precipitation on Yungui Plateau, which ranges from 1,500 to 1,700 mm, further weakened the influence of precipitation across this study.

As a rough forecasting method, this study cannot determine the precise timeline of future change and can only speculate on direction of shift in distribution. The rate of future climate change will be hundreds of times faster than the rate in the past century (Lee et al., 2016). Global and regional ecosystems are predicted to experience rapid change to a drier and warmer future (Guan, 2009).

A limitation of this study is the accuracy with which the percentage of *Pinus* can be inferred in the pollen assemblages (>30%); this is achieved by estimating quantitative standard using power regression. Although it is relatively consistent, the complex dispersal characters of *Pinus* may cause bias in reconstructing accurate relative abundances at sites. Further, in this study we ignored other minor *Pinus* species, such as *P. tabulaeformis and P. massoniana* in the Qinba Mountains, and *P. yunnanensis* and *P. densata* on the Yungui Plateau, which were also present in the study areas. However, we argue here that the approach we have taken in this analysis can be used to understand change in the distribution dominant tree species under the effects of climate change. If the pollen assemblages of sites can be used to accurately reconstruct the past vegetation, such as *Larix, Picea* and *Juglans* (Xu et al., 2007), the results can be rigorous.

## Conclusions

The relationship of the pollen percentage of *Pinus* and the corresponding plant cover reveals that *P. armandii* has been experiencing a range contraction within the study area. Increasing temperature is the main correlate of this change, and we predict that a drier future will continue to contribute to further shifts. *P. armandii* in high-elevation districts tend to disperse in a downhill direction to lower-elevation locations. Regions of lower temperature and lower altitude will be characteristic of *P. armandii* habitat in the future.

##  Supplemental Information

10.7717/peerj.3807/supp-1 Text S1 Supplementary materials

10.7717/peerj.3807/supp-2 Data S1 Raw data collected and used in this article

## References

[ref-1] Aber J, Neilson RP, McNult S, Lenihan JM, Bachelet D, Drapek RJ (2001). Forest processes and global environmental change: predicting the effects of individual and multiple stressors we review the effects of several rapidly changing environmental drivers on ecosystem function, discuss interactions among them, and summarize predicted changes in productivity, carbon storage, and water balance. BioScience.

[ref-2] Beckage B, Osborne B, Gavin DG (2008). A rapid upward shift of a forest ecotone during 40 years of warming in the Green Mountains of Vermont. Proceedings of the National Academy of Sciences of the United States of America.

[ref-3] Box EO (1981). Predicting physiognomic vegetation types with climate variables. Vegetatio.

[ref-4] Canham CD, Thomas RQ (2010). Frequency, not relative abundance, of temperate tree species varies along climate gradients in eastern North America. Ecology.

[ref-5] Ellenberg H (1988). Vegetation ecology of central Europe.

[ref-6] Faegri K, Kaland PE, Krzywinski K (1989). Textbook of pollen analysis.

[ref-7] Giorgi F, HurrellJ W, Marinucci MR, Beniston M (1997). Elevation dependency of the surface climate change signal: a model study. Journal of Climate.

[ref-8] Groom QJ (2013). Some poleward movement of British native vascular plants is occurring, but the fingerprint of climate change is not evident. PeerJ.

[ref-9] Guan L (2009). Preparation of future weather data to study the impact of climate change on buildings. Building and Environment.

[ref-10] Heikkinen RK, Luoto M, Araújo MB, Virkkala R, Thuiller W, Sykes MT (2006). Methods and uncertainties in bioclimatic envelope modelling under climate change. Progress in Physical Geography.

[ref-11] Hirota M, Holmgren M, Van Nes EH, Scheffer M (2011). Global resilience of tropical forest and savanna to critical transitions. Science.

[ref-12] Hutchinson GE (1957). A Treatise on. Limnology. I. Geography, physics, and chemistry.

[ref-13] Kelly AE, Goulden ML (2008). Rapid shifts in plant distribution with recent climate change. Proceedings of the National Academy of Sciences of the United States of America.

[ref-14] Lee O, Park Y, Kim ES, Kim S (2016). Projection of Korean probable maximum precipitation under future climate change scenarios. Advances in Meteorology.

[ref-15] Li WY, Yao ZJ (1990). A study on the quantitative relationship between *Pinus* pollen in surface sample and *Pinus* vegetation. Acta Botanica Sinica.

[ref-16] Luo CX, Zheng Z, Tarasov P, Pan AD, Huang KY, Beaudouin C, An FZ (2009). Characteristics of the modern pollen distribution and their relationship to vegetation in the Xinjiang region, northwestern China. Review of Palaeobotany and Palynology.

[ref-17] Malhi Y, RobertsJ T, Betts RA, Killeen TJ, Li W, Nobre CA (2008). Climate change, deforestation, and the fate of the Amazon. Science.

[ref-18] McLane SC, Aitken SN (2012). Whitebark pine (*Pinus albicaulis*) assisted migration potential: testing establishment north of the species range. Ecological Applications.

[ref-19] Minckley T, Whitlock C (2000). Spatial variation of modern pollen in Oregon and southern Washington, USA. Review of Palaeobotany and Palynology.

[ref-20] Olson DM, Dinerstein E, Wikramanayake ED, Burgess ND, Powell GV, Underwood EC, Loucks CJ (2001). Terrestrial ecoregions of the world: a new map of life on earth a new global map of terrestrial ecoregions provides an innovative tool for conserving biodiversity. BioScience.

[ref-21] Parmesan C (2006). Ecological and evolutionary responses to recent climate change. Annual Review of Ecology, Evolution, and Systematics.

[ref-22] Pearson RG, Dawson TP, Berry PM (2002). SPECIES: a spatial evaluation of climate impact on the envelope of species. Ecological Modelling.

[ref-23] Pearson RG, Thuiller W, Araújo MB, MartinezMeyer E, Brotons L, McClean C, Miles L, Segurado P, Dawson TP, Lees DC (2006). Model-based uncertainty in species range prediction. Journal of Biogeography.

[ref-24] Peñuelas J, Filella I (2001). Responses to a warming world. Science.

[ref-25] R Core Team (2017). R: a language and environment for statistical computing.

[ref-26] Ren GY, Guo J, Xu MZ, Chu ZY, Zhang L, Zou XK, Liu XN (2005). Climate changes of China’s mainland over the past half century. Acta Meteorologica Sinica.

[ref-27] Root TL, Price JT, Hall KR, Schneider SH, Rosenzweig C, Pounds JA (2003). Fingerprints of global warming on wild animals and plants. Nature.

[ref-28] Song DX, Yan JP, Ma L (2011). Study on climatic differentiation in the south and north Qinling Mountains in recent 50 years. Arid Zone Research.

[ref-29] Ter Braak CJF, Smilauer P (2002). http://canoco5.com/.

[ref-30] Tewksbury JJ, Sheldon KS, Ettinger AK (2011). Moving farther and faster. Nature Climate Change.

[ref-31] Toledo M, Peña-Claros M, Bongers F, Alarcón A,  Balcázar J, Chuviña J, Poorter L (2012). Distribution patterns of tropical woody species in response to climatic and edaphic gradients. Journal of Ecology.

[ref-32] Walther GR, Post E, Convey P, Menzel A, Parmesan C, Beebee TJ, Bairlein F (2002). Ecological responses to recent climate change. Nature.

[ref-33] Wang ZY, Ding YH, He JH, Yu J (2004). An updating analysis of the climate change in China in recent 50 years. Acta Meteorologica Sinica.

[ref-34] Xu Q, Li Y, Yang X, Zheng Z (2007). Quantitative relationship between pollen and vegetation in northern China. Science in China Series D.

[ref-35] You WH, He DM, Duan CC (2005). Climate change of the longitudinal range-gorge in Yunnan and its influence on the river flow. Acta Geographica Sinica.

[ref-36] Zhang Y, Kong Z, Wang G, Ni J (2010). Anthropogenic and climatic impacts on surface pollen assemblages along a precipitation gradient in north eastern China. Global Ecology and Biogeography.

