# Supplementary material for: The distribution shifts of Pinus armandii and its response to temperature and precipitation in China
Source: PeerJ. 2017 Sep 15;5:e3807. doi: 10.7717/peerj.3807 (PMC5602682; doi:10.7717/peerj.3807)
Supplement: Text S1 [file peerj-05-3807-s001.docx]

**APPENDIX**

**Qinba Mountains**

1. ***Fargesia spathacea*--*Pinus armandi* forest region**

**Elevation range:** 1700-2500m

**Traits:** grow well, most common *Pinus armandi* forest type, shrub layer coverage larger than 70%

**Associated or mixed arbor tree species:** *Picea asperata,* *Abies fabri, Betula platyphylla*var*. szechuanica, Pinus tabulaeformis, Pinus massoniana, Carpinus cordata, Acer ginnala, Carpinus londoniana*

**Associated or mixed shrub species:** *Bashania fangiana, Yushania chungii, Sorbus koehneana, Rosa omeiensis, Ribes tenue, Fargesia spathacea*

1. **Shrub--*Pinus armandi* forest region**

**Elevation range:** 1500-2300m

**Traits:** high forest land productivity, small in area, shrub layer coverage larger than 50%

**Associated or mixed arbor tree species:** *Tsuga chinensis, Crataegus pinnatifida, Dendrobenthamia japonica*var*. chinensis, Ailanthus altissima, Rhus chinensis, Pinus tabulaeformis, Pinus massoniana*

**Associated or mixed shrub species:** *Viburnum*spp.*, Corylus ferox, Cotoneaster acutifolius, Berberis*sp.

1. **Sparse shrub--*Pinus armandi* forest region**

**Elevation:** 1400-1900m

**Traits:** dominate tree species, sparse shrub layer, grow well but unstable, no regeneration under forest canopy

**Associated or mixed arbor tree species:** *Tilia tuan*var. *chinensis, Betula luminifera, Quercus aliena, Tsuga chinensis, Euptelea pleiospermum, Acer*spp., *Carpinus londoniana, Pinus tabulaeformis, Pinus massoniana*

**Associated or mixed shrub species:** *Lyonia avalifolia*var. *alliptica, Myrica nana, Lonicera*sp., *Euonymus*sp*.*

**Yungui Plateau**

1. **Dense shrub--*Pinus armandi* forest region**

**Elevation:** 1800-2900m, half shady slopes and shady slopes

**Traits:** grow well, most common forest type, canopy density larger than 0.7, sparse shrub layer coverage larger than 50% with complex species component

**Associated or mixed arbor tree species:** *Pinus densata, Tsuga dumosa, Picea likiangensis, Picea brachytyla*var*. complanata, Qurcus longispica, Quercus aquifolioidis, Betula*spp., *Acer*spp., *Castanopsisplatyacantha, Cyclobalanopsis*sp., *Quercus aliena, Q. variabilis, Carpinus turczaninowii,Magnoliceae*spp., *Lauraceae s*pp.

**Associated or mixed shrub species:** *Myrsine ofricana, Rhododendron spinuliferum, Hypericum batutum, Eurya cavinerivis, Lyonia ovalifolia, Viburum cylindricum, Rhododendron spiciferum,*

*Rhododendron decorum, Nothopanax delavayi, Michelia yunnanensis*

1. **Weed--*Pinus armandi* forest region**

**Elevation:** 1400-2900m, sunny slopes

**Traits:** aridity, sparse drought-enduring shrub with the coverage under 5%, artificial *Pinus armandi* forest included

**Associated or mixed arbor tree species:** *Pinus densata, Tsuga dumosa, Picea likiangensis, Picea brachytyla*var. *complanata, Qurcus longispica, Quercus aquifolioidis, Betula*spp*., Acer*spp.*, Lithocarpus  hancei, Lithocarpus dealbatus, Castanopsis delavayi, Quercus aliena, Quercus variabilis, Alnus nepatensis*

**Associated or mixed shrub species:** *Pyracantha fortuneana, Rhododendron decorum, Pyrus pashia, Elaeagnus umbellate, Coriaria sinica*
